# Ibuprofen Blunts Ventilatory Acclimatization to Sustained Hypoxia in Humans

**DOI:** 10.1371/journal.pone.0146087

**Published:** 2016-01-04

**Authors:** Kemal Erdem Basaran, Michael Villongco, Baran Ho, Erika Ellis, Rachel Zarndt, Julie Antonova, Susan R. Hopkins, Frank L. Powell

**Affiliations:** 1 Division of Physiology, Department of Medicine, University of California San Diego, San Diego, California, United States of America; 2 Department of Medical Physiology, Faculty of Medicine, Erciyes University, Melikgazi, Kayseri, Turkey; 3 Department of Radiology, University of California San Diego, San Diego, California, United States of America; University of Colorado Denver, UNITED STATES

## Abstract

Ventilatory acclimatization to hypoxia is a time-dependent increase in ventilation and the hypoxic ventilatory response (HVR) that involves neural plasticity in both carotid body chemoreceptors and brainstem respiratory centers. The mechanisms of such plasticity are not completely understood but recent animal studies show it can be blocked by administering ibuprofen, a nonsteroidal anti-inflammatory drug, during chronic hypoxia. We tested the hypothesis that ibuprofen would also block the increase in HVR with chronic hypoxia in humans in 15 healthy men and women using a double-blind, placebo controlled, cross-over trial. The isocapnic HVR was measured with standard methods in subjects treated with ibuprofen (400mg every 8 hrs) or placebo for 48 hours at sea level and 48 hours at high altitude (3,800 m). Subjects returned to sea level for at least 30 days prior to repeating the protocol with the opposite treatment. Ibuprofen significantly decreased the HVR after acclimatization to high altitude compared to placebo but it did not affect ventilation or arterial O_2_ saturation breathing ambient air at high altitude. Hence, compensatory responses prevent hypoventilation with decreased isocapnic ventilatory O_2_-sensitivity from ibuprofen at this altitude. The effect of ibuprofen to decrease the HVR in humans provides the first experimental evidence that a signaling mechanism described for ventilatory acclimatization to hypoxia in animal models also occurs in people. This establishes a foundation for the future experiments to test the potential role of different mechanisms for neural plasticity and ventilatory acclimatization in humans with chronic hypoxemia from lung disease.

## Introduction

The body’s first line of defense with decreased oxygen levels is the hypoxic ventilatory response (HVR), which is a reflex increase in breathing in response to low arterial partial pressure of oxygen (P_O2_) stimulating carotid body (arterial) chemoreceptors [1]. If hypoxia is sustained, there is a time-dependent increase in both ventilation and the ventilatory sensitivity to acute changes in O_2_ (i.e. the HVR), which collectively are termed “ventilatory acclimatization to hypoxia, or VAH” [1,2]. VAH is observed as early as 24 to 48 hours after ascent to high altitude in healthy humans and persists for at least 8 weeks at an altitude of 3,800m [3–6]. VAH is generally thought to be advantageous because it can increase arterial O_2_ levels over time at a given altitude [1, 2]. This could be beneficial for patients with chronic hypoxemia from lung disease but it is is not clear if VAH occurs in such patients, or if individual variation in VAH contributes to differences in the progression of lung disease. Hence, there is an interest in determining the mechanisms of VAH in humans.

The physiological mechanisms of VAH are not completely understood but considerable progress has been made in animal studies [1]. Sustained hypoxia increases the isocapnic HVR by increasing both carotid body chemoreceptor O_2_-sensitivity, and the “central nervous system gain of the HVR”, which is an increase in ventilatory motor output for a given afferent input from carotid body chemoreceptors to respiratory centers in brain [7,8]. The cellular basis for these changes in animal models is summarized in the Discussion but this study is focused on the molecular signals for such plasticity, which appear to depend on inflammation. Chronic hypoxia increases cytokine gene expression and O_2_-sensitivity in carotid bodies of rats and these changes are blocked by ibuprofen, a non-steroidal anti-inflammatory drug (NSAID) [9]. Ibuprofen administered during chronic hypoxia also blocks VAH in rats [10]. Considering that ibuprofen is a generally safe over-the-counter NSAID, and that it is commonly used by people in sustained hypoxia to alleviate headache from acute mountain sickness, we decided to test the effects of ibuprofen on VAH in healthy humans. Our goal was to determine if NSAID-sensitive signals for VAH described for animal models also apply to people.

## Materials and Methods

### Study Design

We tested the hypothesis that ibuprofen blocks the increase in HVR from chronic hypoxia in healthy humans using a double-blind, placebo controlled, cross-over trial. This study was approved by The UCSD Human Subjects Protection Program. Subjects were recruited by flyers and word of mouth and provided informed written consent. The isocapnic HVR was chosen as the primary outcome because it is the accepted physiological measure of the mechanisms that are sensitive to ibuprofen in the animal experiments that inspired this study, namely O_2_-sensitivity of carotid body chemoreceptors and ventilation. Because the isocapnic HVR can vary in an individual over time [11, 12] (for example during the month we used to insure there were no effects of sustained hypoxia between treatments), we compared the effect of high altitude against a sea level measurement collected with each treatment.

We used the same cohort of subjects in a repeated measures design to improve statistical power. Subjects were randomized to two groups with crossover; one group (n = 7) took placebo capsules during the first half of the study and ibuprofen capsules during the second half, and vice versa for the other group (n = 8). Ibuprofen was administered in the standard over-the-counter dosage of 400 mg every 8 hours. Placebo treatment used identical capsules (#12 caps) with lactose fill. The University of California, San Diego Medical Center Investigational Drug Service prepared both treatments and randomized the treatment order. Treatment order was blind to investigators performing the experiments but known by a physician involved in the experimental design and analysis but not data collection. For each half of the study, subjects were instructed to take the study pill three times per day for two days at sea level and at altitude, with measurements of their HVR made 24 and 48 hours after starting drug or placebo treatment. Investigators insured that subjects had their pills available at the appropriate starting time and checked that subjects took their pills during the measurements at high altitude where they were in residence together. However, drug levels were not verified with blood sampling.

Sea level measurements were performed at the University of California, San Diego and high altitude measurements were performed in the Pace Laboratory at the Barcroft Facilities of the White Mountain Research Station (3,800m above sea level, P_O2_ = 90 Torr). We acclimatized subjects for 48 hrs because most of the increase in the isocapnic HVR happening over 2 months at this altitude occurs by this time [4–6]; we studied 24 hrs also to obtain more data and test for time-dependent effects of ibuprofen at sea level. After completion of the first half of the study, subjects returned to sea level for one month to insure deacclimatization. The protocol was then repeated with measurements at sea level and at altitude, with each subject taking the other study pill.

The data for this study was collected over three summers because of logistical constraints working at the high altitude laboratory, which is only open during summer months, and challenges in recruiting subjects who could be away from work or school for several days at a time in successive months. However, the additional statistical power of the repeated measures design outweighed these challenges. At the end of the first year, after all of the data had been analyzed and validated, we un-blinded the treatment to test for effects of ibuprofen on altitude responses. Results from these 5 subjects showed a trend for ibuprofen to reduce the effect of altitude to increase the HVR and power calculations based on the observed variability indicated we would need to study 22 subjects to demonstrate significance. After 3 years, we had collected complete data on 15 subjects but the cross-over design was randomized evenly in these subjects and we found a significant effect (see Results) so we ended the study.

### Subjects

Complete data sets were obtained for 9 males and 6 females between the ages of 18 and 26 years (average = 24), with a body mass ranging from 45.3 to 94.3 kg, average 63.0 ± 12.3 kg SD. A medical history was taken and a physical examination performed with special reference to the cardiopulmonary system to insure subjects were free of cardiovascular or respiratory disease and had no history of allergies to NSAIDs.

### HVR protocol

We used a protocol we have developed over several years for measuring the steady state, isocapnic HVR, which is generally accepted as a reflex measurement of the hypoxic sensitivity of carotid bodies and ventilation [1]. Our method [6] is as a modification of protocols used by the Severinghaus laboratory [4, 5], which was designed to quantify the isocapnic HVR at different points in time during sustained hypoxia and account hypoxic ventilatory decline (HVD). HVD is defined as the decrease in ventilation that starts to occur in the first minutes of sustained hypoxia [2]. HVD is primarily a decrease in ventilation without a change in the ventilatory O_2_-sensivity, although there is evidence for some decrease in ventilatory O_2_-sensitivity over the first few minutes of hypoxia [13].

Our protocol has been described in detail before [6, 13]. Subjects underwent a screening session one week prior to the first experiment to familiarize them with breathing through the mask and to insure they had no adverse responses to hypoxia. An EKG was measured to insure that subjects had no arrhythmias or other abnormal cardiac changes during acute hypoxia. The time on the breathing apparatus (ca. 30 min) was less than during the full protocol and typically this screening was completed on the same day as taking a history and making physical exam.

For experimental sessions, subjects were seated in a semi-recumbent position listening to music of their choice and breathed through a face mask (Hans Rudolph, Model 7450 V2) connected to a partial rebreathing circuit, which facilitated control of end-expired P_CO2_ (Pe’_CO2_) while changing inspired O_2_ levels [4]. A three-way valve directed either room air or a mixture of O_2_/N_2_/CO_2_ (Matheson Tri-Gas, MFMR-1138-SA) to the inspiratory port of a non-rebreathing valve (Hans Rudolph, 2700 2WNRBV). Subjects started breathing room air for 5 minutes to become comfortable on the equipment, as evidenced by a regular breathing pattern and stable Pe'_CO2_ (± 2 Torr). Then we switched the three-way valve and subjects started breathing gas mixtures administered in the rebreathing circuit. Subjects breathed a mild hyperoxic gas mixture (Pi_O2_ ≈ 210mmHg, equivalent to 30% O_2_ at sea level) for 15 minutes to raise Sa_O2_ to 100% at both sea level and altitude, and to reverse any potential HVD that can occur with ambient hypoxia at high altitude [4].

Next we established the isocapnic end-expired P_CO2_ level to be maintained throughout HVR measurements (iPe’_CO2_) by increasing inspired CO_2_ until Pe’_CO2_ was 2–3 Torr above the hyperoxic level. This will slightly increase the isocapnic HVR by the well-known multiplicative interaction between O_2_ and CO_2_ as carotid body stimuli, but more importantly it allows us to maintain isocapnia even if HVD should decrease ventilation, and increase Pe’_CO2_ during the protocol [4]. These conditions were maintained for 5 minutes and then we rapidly deceased inspired O_2_ until Sa_O2_ reached a target value of 90% and maintained this level for 5 minutes. The measured change in ventilation between Sa_O2_ = 100% and 90% quantified the acute HVR without any HVD. We termed this HVR1 as the first measurement of ventilatory O_2_-sensitivity in the protocol, which was expressed as the increase in $\dot{V}$i per percent decrease in Sa_O2_ [1, 14].

Then we quantified changes in the HVR with HVD by measuring the HVR during two additional steps to deeper hypoxia (Sa_O2_ = 80%) at 8 and 14 minutes after the first decrease in Sa_O2_ to 90%. These deeper bouts of hypoxia lasted only 3 min and the change in ventilation between Sa_O2_ = 90% and 80% was used to calculate an HVR2 and HVR3, as the second and third measurements of HVR in our protocol [6, 13].

Finally, after finishing HVR measurements, subjects returned to breathing Pi_O2_ ≈ 210 Torr for 10 minutes to recover from the hypoxia. Then we measured the hypercapnic ventilatory response (HCVR) by increasing Pe’_CO2_ 6 to 10 Torr above the newly measured Pe’_CO2_ value for 6 min [4]. HCVR was expressed as the increase in $\dot{V}$i per Torr increase in Pe’_CO2_.

### Physiological Measurements

The primary physiological variables were inspired ventilation ($\dot{V}$i) measured with a pheumotachograph (Fleisch #3) coupled to a differential pressure transducer (Validyne, MP45 ±2 cmH_2_O) and arterial O_2_ saturation (Sa_O2_) monitored continuously with a forehead sensor connected to a pulse oximeter (Nellcor, N395), which has been validated against arterial blood in our laboratory [15]. End-expired P_O2_ and P_CO2_ were measured in the mouthpiece continuously with gas analyzers (Beckman OM11 and LB-2). Analog signals for these measurements were fed to a data acquisition system (BIOPAC Systems, Inc., MP100A) and imported into data acquisition software (BIOPAC Systems, Inc., AcqKnowledge version 3.9.1.6, build 06292007) for subsequent analysis of $\dot{V}$i, (Lbtps/min), tidal volume (Vt mlbtps), fr (breaths/min) end-expired P_CO2_ (Pe’_CO2_ Torr), estimated arterial O_2_ saturation (Sa_O2_%) and to calculate the HVR and HCVR. HVD was calculated as the decrease in $\dot{V}$i predicted for Sa_O2_ = 85% between HVR2 and HVR 3 measurements (8 and 14 minutes after the start of hypoxia).

### Statistical Analysis

We used ANOVA for repeated measures (Statview, 5.0.1 SAS Institute, Cary, NC) to test for significant effects of (1) drug, 2 levels = placebo, ibuprophen, (2) altitude, 2 levels = sea level, 3800 m, and (3) time, 2 Levels = 24 hrs, 48 hrs. When we found significant main effects or interactions, we did post hoc testing with Fisher’s Protected Least Significant Difference. The null-hypothesis (no effect) was rejected for P < 0.05, using two tailed for effects on HVD one tailed for variables with known effects on physiological variables (e.g. altitude increases HVR and decreases Sa_O2_), or to test our hypothesis that drug treatment with ibuprofen decreases the HVR at altitude. Data are reported as the mean ± standard deviation.

## Results

Twenty-four subjects were screened and signed the consent form but six subjects withdrew from the study because of scheduling conflicts and three subjects did not produce usable data (one had no measurable or a negative HVR, another became nervous and showed extreme variability in breathing independent of respiratory stimuli and one exercised too hard during one of the trips to altitude and became ill). Some of the remaining 15 subjects had symptoms of acute mountain sickness but no one felt ill enough to withdraw from the study.

**Fig 1:**
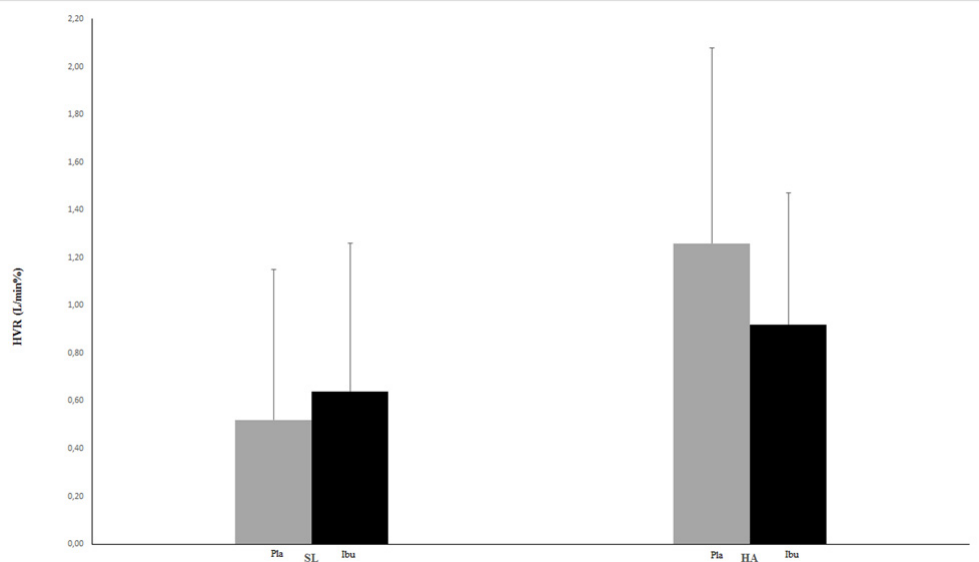
Effects of altitude and ibuprofen on the hypoxic ventilatory response (HVR = Δ$\dot{V}$i / ΔSa_O2_). Average HVR1 (± SD), which is the initial measure of the acute HVR, is plotted for 48 hrs of treatment with placebo (Pla, gray bars) or ibuprofen (Ibu, black bars) at sea level and high altitude. There was a significant effect of altitude (p = 0.001) and altitude by drug interaction (p = 0.03). Post hoc analysis showed all of the values were significantly different from each other (p < 0.05) except the two sea level values (p = 0.22).

Fig 1 shows the effect of ibuprofen versus placebo on the HVR before and after acclimatization to high altitude. It plots HVR1 at 48 hrs, which measures ventilatory sensitivity to O_2_ with CO_2_ held constant before there is any hypoxic ventilatory decline. With placebo, HVR1 increased an average of 2.4-fold at altitude relative to sea level but it only increased 1.4-fold with ibuprofen. ANOVA showed a significant altitude by drug interaction (Table 1). Post hoc testing showed that although high altitude increased HVR1 significantly with either placebo (p = 0.0001) or ibuprofen (p = 0.035), the increase in HVR1 was significantly less with ibuprofen versus placebo at high altitude (p = 0. 015). HVR1 was not affected by ibuprofen at sea level (p = 0.22).

**Table 1:** Effects of placebo vs. ibuprofen treatment on ventilatory acclimatization to altitude. Average values (±S.D.) and effects of placebo vs. ibuprofen treatment for 2 days on respiratory variables at sea level and altitude. P values are for ANOVA tests of an altitude by drug interaction and significant values (p < 0.05) indicate the change in a variable between sea level and altitude is significantly different with ibuprofen vs. placebo. All variables except $\dot{V}$I showed a significant effect of altitude (p < 0.05). Sa_O2_, arterial O_2_ saturation measured by pulse oximeter breathing ambient air (%); Pe’_CO2_, end-expired P_CO2_; iPe’_CO2_, isocapnic end-expired P_CO2_; $\dot{V}$I, ventilation breathing ambient air (L/min); HVR1, HVR2 and HVR3, hypoxic ventilatory response measured between Sa_O2_ = 100% and 90% (L /(min %)); HVD, hypoxic ventilatory decline, see text (L/min); HCVR, hypercapnic ventilatory response (L (min/Torr)).

		Placebo				Ibuprofen			
		Sea Level		Altitude		Sea Level		Altitude	
Variable	P	24	48	24	48	24	48	24	48
Sa_O2_ (%)	0.29	98.8±0.8	99.3±0.8	89.3±3.9	88.8±4.4	99.1±1.4	99.2±0.7	90.1±2.3	88.4±3.1
Pe’_CO2_ (Torr)	0.75	35.6±3.1	34.6±2.6	29.0±2.5	28.8±2.3	36.0±3.0	36.4±3.3	29.7±2.4	30.2±2.2
iPe’_CO2_ (Torr)	0.08	38.8±2.7	37.7±2.6	33.6±2.2	33.1±1.8	39.1±2.6	40.1±3.0	33.0±3.3	33.3±1.3
$\dot{V}$i (L /min)	0.38	9.79±1.72	9.61±1.87	11.46±4.81	11.84±3.80	11.31±1.58	10.70±2.23	10.49±4.09	11.40±3.29
HVR1 (L/min %)	0.03	0.47±0.50	0.52±0.63	1.11±0.81	1.26±0.82	0.68±0.54	0.64±0.62	1.06±0.065	0.92±0.55
HVR2 (L/min %)	0.005	0.46±0.48	0.50±0.51	0.96±0.69	0.82±0.51	0.66±0.76	0.78±0.91	0.56±0.30	0.82±0.49
HVR3 (L/min %)	0.03	0.28±0.32	0.47±0.36	0.84±0.61	0.74±0.63	0.63±0.65	0.44±0.36	0.88±0.52	0.57±0.39
HVD (L/min)	0.05	1.36±2.52	1.79±3.95	3.77±3.90	4.88±5.69	4.03±6.22	4.42±7.77	2.57±5.95	5.49±4.68
HCVR (L/min Torr)	0.68	0.92±0.46	1.03±0.72	3.12±2.74	2.85±2.13	1.16±0.62	1.04±0.44	3.04±2.43	2.64±1.18

Similar effects were observed on HVR2 and HVR3 (Table 1), which measure ventilatory O_2_-sensitivity after hypoxic ventilatory decline (HVD) is established during sustained hypoxia in our protocol. Both HVR2 and HVR3 showed significant altitude by drug interactions (Table 1) and decreased with ibuprofen at high altitude, similar to the response shown for HVR1 in Fig 1. There were no significant differences between HVR1, HVR2 and HVR3 in a given condition so HVD did not affect ventilatory O_2_-sensitivity, but absolute levels of $\dot{V}$i decreased with sustained hypoxia during HVR2 and HVR3 measurements. HVD was calculated as the change in $\dot{V}$i predicted for Sa_O2_ = 85% between HVR2 and HVR3 to measurements, which is the decrease in $\dot{V}$i between 8 and 14 minutes of sustained hypoxia. HVD showed a significant altitude by drug interaction (p = 0.047, Table 1) and post hoc analysis of the 48 hour HVD points showed significant increases at high altitude with placebo (p = 0.009) or ibuprofen (p = 0.03) versus placebo at sea level. There was a non-significant trend (p = 0.06) for ibuprofen to increase HVD at sea level compared to placebo.

Table 1 summarizes the other respiratory variables for sea level and high altitude during treatment with ibuprofen also. There was a significant effect of altitude on all variables except $\dot{V}$i, although it tended to be greater at altitude because Vt increased while fr was essentially constant under all conditions (16.8 ± 0.6 breaths/min). Sa_O2_ was about 10% less at high altitude with placebo or ibuprofen. Hence, there was no significant altitude by drug interaction for $\dot{V}$i or Sa_O2_, in contrast to the significant effect on the HVR (Table 1). The decrease in isocapnic ventilatory O_2_-sensitivity with ibuprofen at high altitude must be offset by other factors that prevented significant decreases in breathing or arterial oxygenation when Pa_CO2_ was allowed to change during ambient air breathing at this altitude.

Altitude significantly increased the hypercapnic ventilatory response (HCVR, p = 0.0001) but there was no effect of ibuprofen (Table 1). HCVR increased 2.4 and 2.9-fold with placebo and ibuprofen at altitude, respectively and there was no significant drug by altitude interaction to indicate and difference with ibuprofen (Table 1). Pe’_CO2_ decreased similarly with placebo or ibuprofen at altitude also (Table 1). Hence, the effect of ibuprofen at high altitude appears specific to O_2_-sensitivity.

## Discussion

The results support our hypothesis that ibuprofen impairs ventilatory acclimatization to high altitude in healthy humans, similar to the effect described in animal studies [10]. Specifically, the isocapnic HVR after acclimatization to high altitude was significantly decreased by ibuprofen versus placebo (Fig 1). Hence, the time-dependent increase of the ventilatory response to *changes* in O_2_ at high altitude in humans depends on a physiological mechanism that is sensitive to a non-steroidal anti-inflammatory drug. We used the standard state of the art methods to demonstrate these effects [1] and controlled for the potential changes in the HVR that can occur over time within individuals [11, 16] by an experimental design that compared altitude and sea level responses measured independently with both treatments.

Our results also show that despite effects of ibuprofen on ventilatory O_2_-sensitivity, other mechanisms compensate to prevent decreases in breathing and arterial oxygenation during 48 hrs at this altitude. This is similar to previous studies on the effects of ibuprofen as a treatment for headache with acute mountain sickness (AMS). Gertsch and co-workers [17] found no significant difference in Sa_O2_ with ibuprofen versus placebo in 183 subjects who completed their double-blind, randomized, placebo-controlled trial. This excluded subjects who had to withdraw for severe AMS, so it is a population similar to that we studied.

Similar $\dot{V}$i and Sa_O2_ with ibuprofen treatment in our study is also consistent with the well-known multiple elements of ventilatory acclimatization to hypoxia (VAH) with distinct mechanisms. Acclimatization to hypoxia increases both (i) the slope of hypoxic ventilatory response curves ($\dot{V}$i vs. Sa_O2_) and (ii) the intercept, i.e. $\dot{V}$i when Sa_O2_ = 100%. The latter effect manifests as persistent hyperventilation when normoxia is restored after sustained hypoxia, and has been termed “ventilatory deacclimatization to hypoxia” to emphasize it is not simply the “off response” of VAH but a distinct mechanism [2]. Experiments show that ventilatory O_2_-sensitivity can change independently of the persistent hyperventilation in normoxia after chronic hypoxia (cf. [2]). While it is generally accepted that increased ventilatory O_2_-sensitivity in VAH results from both increased carotid body O_2_-sensitivity and sensitivity of central respiratory centers to chemoreceptor afferent input, it is not clear if either of these mechanisms can explain ventilatory deacclimatization hypoxia [18]. However, we note that whatever compensatory effects may be occurring with ibuprofen at high altitude, there could be different effects on $\dot{V}$i and Sa_O2_ if subjects were exposed to deeper levels of hypoxia. For example, ibuprofen significantly decreases $\dot{V}$i in rats breathing 10% O_2_ without isocapnia [10], which is similar to an altitude of 6,000m above sea level.

The fact that we observed similar effects of ibuprofen at altitude on HVR measured after different durations of hypoxia (i.e. HVR1, 2 and 3) suggests that the ibuprofen effect on O_2_-sensitivity is independent of hypoxic ventilatory decline (HVD). HVD is a decrease in ventilation during the first minutes of sustained hypoxia and it can occur without a significant decrease in ventilatory sensitivity to changes in O_2_, i.e. it is mainly a decrease in the intercept of a plot of $\dot{V}$i vs. Sa_O2_ without a change in slope [4,13]. However, our data cannot rule out an independent effect of ibuprofen on HVD. There was a significant altitude by drug interaction for HVD (p = 0.05) and HVD tended to be greater under all conditions compared to placebo at sea level (Table 1). High altitude exposure for 48 hrs significantly increased HVD (relative to the 48 hr sea level placebo value) by 2.7-fold with placebo (p = 0.009) or 3.1-fold with ibuprofen (p = 0.03). This effect of 48 hrs of acclimatization on HVD is consistent with some studies [5] but not others [13] so it is difficult to draw any general conclusions. Our data also showed a nearly significant (p = 0.06) 2.5-fold increase in HVD after 48 hrs of ibuprofen at sea level compared to placebo. The power of our study is insufficient to reliably rule out such a potential effect of ibuprofen on HVD at sea level and there is no other evidence published about this so it needs further study.

Finally, the results show that the effects of ibuprofen are specific to O_2_-sensitivity because there were no significant effects of the drug on the hypercapnic ventilatory response (HCVR) (Table 1). End-expired P_CO2_ values were comparable under different conditions in our experiments (Table 1) so well-known interactions between O_2_ and CO_2_-sensitivity for chemoreceptors and ventilation [1] cannot explain the observed differences. We did not measure arterial blood gases in our study but animal studies show no effects of ibuprofen on arterial pH independent of any changes in arterial P_CO2_ [10], so there is no evidence for ibuprofen-dependent effects on pH that could affect ventilation.

### Physiological Significance

The primary goal of our study was to determine if a specific physiological mechanism for ventilatory acclimatization to hypoxia that has been described in animals also occurs in humans. This mechanism involves neuroimmune signals that are analogous to those induced in the peripheral nervous system during chronic inflammatory pain, and which also cause chemoreceptor plasticity [19]. In rats, chronic sustained hypoxia increases the number of ED1+ immune cells in the carotid body and the expression of mRNA for cytokines; the expression of cytokine genes was not limited to immune cells but also involves carotid body Type 1 and 2 cells [9]. These changes and the increased hypoxic neural responses of carotid body chemoreceptors with chronic hypoxia could be blocked by dexamethasone or ibuprofen [9]. The ibuprofen dose used for those animal studies (4 mg/kg) was chosen for its ability to suppress phenotypic changes in rat primary sensory neurons induced by inflammation [20] and was equivalent to the therapeutic dose for a child. That same ibuprofen dose also blocks the effect of chronic hypoxia to increase cytokine gene expression in medullary respiratory centers and ventilatory acclimatization to hypoxia in rats [10]. In this study, we used a dose of 400 mg, 3 times/day for all subjects, resulting in a dose of ca. 6 mg/kg 3 times/day, which is at the low end of the therapeutic range for adults (400–800 mg, 3–4 times/day). As discussed above, ibuprofen is frequently used at such doses to treat high altitude headache without affecting respiration. The observation that similar doses of ibuprofen block ventilatory acclimatization to hypoxia in both rats and humans supports our hypothesis that sustained hypoxia evokes the same mechanism involving inflammatory signals in both species.

However, questions remain about the exact mechanisms of the ibuprofen on VAH in humans and answering them will be important to understand if neural plasticity in ventilatory control plays a role in patients with chronic hypoxemia. Progress is being made on the cellular mechanisms and molecular signals for such plasticity in animal studies. These show that neural plasticity with chronic hypoxia involves plasticity that is sensitive to ibuprofen in both peripheral arterial (carotid body) chemoreceptors [9] and respiratory centers in the central nervous system (CNS) [10]. Cytokines expressed in chronically hypoxic carotid bodies, which can be blocked by ibuprofen, cause increased gene expression for chemical and voltage sensitive ion channels that increase O_2_-sensitivity [19]. Plasticity in CNS respiratory centers with chronic sustained hypoxia involves changes in glutamatergic neurotransmission, ion channels that increase synaptic transmission and intrinsic neuronal excitability, and neuron-glia interactions [21–23]. Glia are an important source of cytokines in neuropathic pain, which is hypothesized to share common mechanisms with carotid body acclimatization to hypoxia and is sensitive to ibuprofen [19,24, 25]. Finally, while ibuprofen is a cyclooxygenase (COX) inhibitor, it may act by mechanisms other than its classic anti inflammatory effect to reduce prostaglandin formation by inhibiting COX-2 [26]. Ibuprofen can block NF-κB transcription of cytokines independently of its effect to inhibit COX [27]. Also, hypoxia increases NF-κB both directly via prolyl hydroxylases [28], and indirectly through positive feedback interactions between NF-κB and hypoxia inducible factor-1**α** α (HIF-1**α**) [29–32]. HIF-1**α** is neccessary for normal VAH [33] so ibuprofen has the potential to affect VAH via NF-κB effects on both cytokines and HIF-1**α**. Further study will be necessary to determine which, if any, of these cellular mechanisms and molecular signals are involved in VAH in humans in health or disease.
